# Preconditioners for the geometry optimisation and saddle point search of molecular systems

**DOI:** 10.1038/s41598-018-32105-x

**Published:** 2018-09-18

**Authors:** Letif Mones, Christoph Ortner, Gábor Csányi

**Affiliations:** 1 Mathematics Institute, University of Warwick, Zeeman Building, Coventry, CV4 7AL United Kingdom; 2 Engineering Laboratory, University of Cambridge, Trumpington Street, Cambridge, CB2 1PZ United Kingdom

## Abstract

A class of preconditioners is introduced to enhance geometry optimisation and transition state search of molecular systems. We start from the Hessian of molecular mechanical terms, decompose it and retain only its positive definite part to construct a sparse preconditioner matrix. The construction requires only the computation of the gradient of the corresponding molecular mechanical terms that are already available in popular force field software packages. For molecular crystals, the preconditioner can be combined straightforwardly with the exponential preconditioner recently introduced for periodic systems. The efficiency is demonstrated on several systems using empirical, semiempirical and ab initio potential energy surfaces.

## Introduction

Geometry optimisation and transition state search are fundamental procedures to identify important stationary points of molecules, molecular crystals and material systems in computational chemistry. Since the evaluation of chemically accurate ab initio potential energies and gradients are computationally demanding, several techniques have been developed over the last three decades to enhance the convergence of optimisation methods.

Among the most widely used are quasi-Newton methods, in particular BFGS or its limited memory version^1^, which start with a (scaled) identity as their guess for the Hessian and update it at each iteration based on the gradient information collected from previous steps. Initialising with a Hessian guess that includes more molecule specific geometrical information can improve the convergence. For instance, just introducing some connectivity information about a molecule can lead to surprisingly good results^2,3^.

Utilising internal coordinates in the construction of Hessian has also been intensively investigated^4–9^. These approaches include (1) methods that build the Hessian matrix in the space of internal coordinates in the beginning of the optimisation and then transform it to Cartesian coordinates, (2) methods that recompute and transform the internal Hessian every step and (3) methods that carry out even the optimisation step in the space of internal coordinates. However, this latter scheme requires to carry out both the projection of Cartesian gradients to internal coordinates gradients and the transformation of the internal coordinates to Cartesian coordinates. None of these steps is straightforward and the gradient conversion can be accomplished only by an iterative solution due to the curvilinear nature of the transformation^8^.

More sophisticated methods estimate the initial Hessian guess from a surrogate potential (e.g. force field or semiempirical potential) whose second derivative can be obtained at low computational cost. The update of the approximate Hessian can then be achieved either by using quasi-Newton methods or other techniques such as DIIS^10,11^. Such strategies significantly improve the speed of convergence in either Cartesian or internal coordinates^6^.

If the model Hessian is cheap to calculate (e.g., as obtained from a surrogate model) and provides a reasonable approximation of the quantum Hessian, it may be advantageous to recompute it at every optimisation step. Such a scheme was introduced by Lindh *et al*.^12^, where a model potential is constructed consisting of quadratic terms for all distances, angles and dihedrals in the molecule. At each geometry optimisation step the force field is constructed such that the current conformation is its local minimum, and its “Hessian” is computed, neglecting the dependence of the force field parameters on the geometry. With this construction, the model “Hessian” is therefore not the Hessian of any potential. Nevertheless, this approach yields excellent performance, which led to its wide implementation in quantum chemistry program packages (e.g. in MOLPRO^13^, ORCA^14^, DALTON^15^, CRYSTAL^16^).

The model “Hessian” of Lindh^12^ can be considered as a preconditioner or metric that effects a transformation to a new coordinate system where the optimisation problem is better conditioned, hence algorithms converge more rapidly and tend to be more robust. Geometrically, the shape of the energy landscape becomes more isotropic. In general it is desirable that both the construction and inversion of the preconditioner matrix are inexpensive (at least compared to the computation of the energy and gradient). This can be achieved by building a sparse preconditioner from simple analytical functions. Since the preconditioner defines a metric in configuration space it needs to be positive definite. This requirement is automatically fulfilled in the Lindh approach^12^ by the use of quadratic molecular mechanical terms with equilibrium points corresponding to the actual geometry at each step.

We recently introduced a general and effective preconditioner for geometry optimisation and saddle point search for material systems^3^. This preconditioner is determined by the local connectivity structure of atoms making both the construction and inversion computationally inexpensive. Especially for larger systems we observe an order of magnitude or larger reduction of the number of optimisation steps for the preconditioned LBFGS method compared to the same without preconditioning. Here we expand our previous work to molecules and molecular crystals by combining it with a force field based preconditioner inspired by the approach of Lindh *et al*.^12^.

## Methods

### Enhancing geometry optimisation by using preconditioners

We briefly review the methodology for preconditioning geometry optimisation and the dimer saddle point search method for material systems^3^. For a system with *N* particles let ${x}_{k}\in {{\mathbb{R}}}^{3N}$ denote the configuration at the *k* th iterate of an optimisation algorithm. The corresponding energy, gradient and preconditioner are denoted by *f*_*k*_ = *f*(*x*_*k*_), *g*_*k*_ = ∇*f*(*x*_*k*_) and ${P}_{k}\in {{\mathbb{R}}}^{3N\times 3N}\approx {\nabla }^{2}f({x}_{k})$, respectively. A preconditioned steepest-descent step is then given by

1 $${x}_{k+1}={x}_{k}-{\alpha }_{k}{P}_{k}^{-1}{g}_{k}$$

where *α*_*k*_ is the step size obtained from some line search procedure at the *k* th iteration. If *P*_*k*_ = *I*, then (1) becomes the standard steepest descent scheme and if *P*_*k*_ = ∇^2^*f*(*x*_*k*_), then it becomes a quadratically convergent Newton scheme. In general, different choices of *P*_*k*_ may “interpolate” between these extremes.

From an alternative point of view preconditioning can be thought of as a coordinate transformation, where a new set of coordinates is defined as ${y}_{k}:={P}^{\mathrm{1/2}}{x}_{k}$. The advantage of this framework is that once an appropriate preconditioner matrix is available then any optimisation algorithm can be modified by applying the original algorithm on the transformed coordinates. To obtain the final form of the modified algorithm we need to transform the variables back to the original coordinate system. As a simple example, applying the coordinate transformation on the gradient descent equation immediately leads to the equation of quasi Newton schemes (eq. 1):

2 $$\begin{array}{rcl}{y}_{k+1} & = & {y}_{k}-{\alpha }_{k}{\nabla }_{y}F({y}_{k})\\ {P}_{k}^{\mathrm{1/2}}{x}_{k+1} & = & {P}_{k}^{\mathrm{1/2}}{x}_{k}-{\alpha }_{k}{P}_{k}^{-\mathrm{1/2}}{\nabla }_{x}\,f({x}_{k})\\ {x}_{k+1} & = & {x}_{k}-{\alpha }_{k}{P}_{k}^{-1}{\nabla }_{x}\,f({x}_{k})\end{array}$$

where we used the fact that ${\nabla }_{y}F({y}_{k})={P}_{k}^{-\mathrm{1/2}}{\nabla }_{x}\,f({x}_{k})$. Preconditioning popular optimisation methods like LBFGS, conjugate gradients, FIRE^17^ etc. is similarly possible.

A simple preconditioner that is effective for a wide range of materials systems is based on the following *N* × *N* matrix^3^

3 $${L}_{ij}=\{\begin{array}{cc}-\mu \exp (\,\,-\,A(\frac{{r}_{ij}}{{r}_{{\rm{n}}{\rm{n}}}}-1)), & i\ne j\,\text{and}\,|{r}_{ij}| < {r}_{{\rm{c}}{\rm{u}}{\rm{t}}}\\ 0, & i\ne j\,\text{and}\,|{r}_{ij}|\ge {r}_{{\rm{c}}{\rm{u}}{\rm{t}}}\\ -\sum _{i^{\prime} \ne j}{L}_{i^{\prime} j}, & i=j\end{array}$$

where *i* and *j* denote atomic indices and *μ*, *A*, *r*_cut_ and *r*_nn_ are parameters that can be user-specified or estimated numerically. We note that (3) is a generalisation of the Laplacian matrix used to represent undirected graphs. Given a specific connectivity defined by *r*_cut_ and setting *A* = 0 and *μ* = 1, *L*_*ij*_ reduces exactly to the Laplacian matrix. The actual 3*N* × 3*N* preconditioner is simply obtained from the corresponding *L*_*ij*_ element in an isotropic manner:

4 $$[{P}_{{\rm{E}}{\rm{x}}{\rm{p}}}{]}_{k+3(i-1),l+3(j-1)}=\{\begin{array}{cc}{L}_{ij}, & k=l\\ 0, & k\ne l\end{array}$$

where the *k* and *l* indices denote Cartesian components. Despite its simplicity in capturing only geometric connectivity but no specific material information, *P*_Exp_ provides a good model for the local curvature of the potential energy landscape, which effectively controls ill-conditioning in large systems. The application of *P*_Exp_ resulted in a significant reduction of the number of optimisation steps required for several material systems compared to the unpreconditioned LBFGS^3^.

### FF-based preconditioners

Preliminary tests showed that for molecular systems such as molecules in gas phase or molecular crystals using *P*_Exp_ still leads to a speed-up, but a much more modest one than for material systems. The explanation for this is that molecular systems contain a wide range of different interactions (e.g., pair, angle, dihedral, electrostatic, dispersive) of vastly varying stiffness which in addition are more loosely coupled, and this creates a second source of ill-conditioning distinct from ill-conditioning due to large system size. Inspired by the use of internal coordinates in molecular optimisation techniques^4^ and the model Hessian of Lindh *et al*.^12^ we therefore propose a generalisation of *P*_Exp_ that is effective also for molecular systems.

The construction of our *FF-based preconditioner* begins with a surrogate potential energy function, given by a sum over internal coordinates each describing a short-range bond in the system (distance, angle, or dihedral),

5 $${V}_{{\rm{FF}}}=\sum _{\alpha }{V}_{\alpha }({\xi }_{\alpha }(x)).$$

The individual potential energy terms are in general simple functions of the internal coordinates. Some examples of most typical forms are the quadratic, Morse or torsional potentials, respectively given by

6 $${V}_{{\rm{Quadratic}}}(q)=\frac{1}{2}k{(q-{q}_{0})}^{2},$$

7 $${V}_{{\rm{Morse}}}(d)={D}_{0}{(1-\exp (-\alpha (d-{d}_{0})))}^{2},$$

and

8 $${V}_{{\rm{Torsion}}}(\varphi )=\frac{1}{2}{k}_{\varphi }(1+\,\cos (n\varphi -{\varphi }_{0}))$$

where the corresponding parameters can be taken from standard force field libraries.

Due to its simple functional form ${H}_{{\rm{FF}}}\,:\,=\,{\nabla }^{2}{V}_{{\rm{FF}}}$ is cheap to compute. If only short-range bonds are taken into account then it is also sparse, hence it is cheap to store and invert. Moreover, we expect that *V*_FF_ gives a good qualitative approximation to the quantum potential energy landscape, hence *H*_FF_ is a good qualitative approximation to the quantum Hessian ∇^2^*f*. Therefore, *H*_FF_ satisfies all the conditions required for a preconditioner *except* that it will in general be indefinite. A conceptually straightforward but computationally expensive approach to overcome this limitation is to enforce positivity by replacing all eigenvalues of *H*_FF_ with their absolute values. Instead, we propose to analytically modify the local Hessian contributions to ensure overall positivity, resulting in a further *reduction* in computational cost.

The Hessian contribution from *V*_*α*_ is given by

9 $${H}_{\alpha }=\frac{{\partial }^{2}{V}_{\alpha }}{\partial {x}^{2}}=\mathop{\underbrace{\frac{\partial {\xi }_{\alpha }}{\partial x}\otimes \frac{\partial {\xi }_{\alpha }}{\partial x}\frac{{\partial }^{2}{V}_{\alpha }}{\partial {\xi }_{\alpha }^{2}}}}\limits_{{H}_{\alpha }^{(1)}}+\mathop{\underbrace{\frac{{\partial }^{2}{\xi }_{\alpha }}{\partial {x}^{2}}\frac{\partial {V}_{\alpha }}{\partial {\xi }_{\alpha }}}}\limits_{{H}_{\alpha }^{(2)}},$$

where we have decomposed *H*_*α*_ into two terms, ${H}_{\alpha }^{\mathrm{(1)}}$ and ${H}_{\alpha }^{\mathrm{(2)}}$. If *V*_*α*_ is quadratic then $\frac{{\partial }^{2}{V}_{{\rm{Quadratic}}}}{\partial {\xi }^{2}}=k > 0$, hence ${H}_{\alpha }^{\mathrm{(1)}}$ is positive semi-definite, while the sign of ${H}_{\alpha }^{\mathrm{(2)}}$ is ambiguous. Note however, that if the system is at equilibrium of *V*_*α*_ with respect to *ξ*_*α*_, i.e., if $\frac{\partial {V}_{\alpha }}{\partial {\xi }_{\alpha }}=0$, then ${H}_{\alpha }^{\mathrm{(2)}}=0$. This in fact is the case in the Lindh approach^12^. Instead of adjusting *V*_*α*_ at every step such that the geometry corresponds to its equilibrium, here we simply drop ${H}_{\alpha }^{\mathrm{(2)}}$ and only use ${H}_{\alpha }^{\mathrm{(1)}}$ to construct the preconditioner, thus ensuring that it always stays positive definite.

For non-quadratic contributions we expect that $\frac{{\partial }^{2}{V}_{\alpha }}{\partial {\xi }_{\alpha }^{2}} > 0$ for most but not all bonds, hence we enforce positivity by replacing it with its absolute value. This leads to the following general preconditioner for molecular systems:

10 $${P}_{{\rm{F}}{\rm{F}}}=\sum _{\alpha }{\mathop{H}\limits^{ \sim }}_{\alpha }^{(1)}=\sum _{\alpha }\frac{{\rm{\partial }}{\xi }_{\alpha }}{{\rm{\partial }}x}\otimes \frac{{\rm{\partial }}{\xi }_{\alpha }}{{\rm{\partial }}x}|\frac{{{\rm{\partial }}}^{2}{V}_{\alpha }}{{\rm{\partial }}{\xi }_{\alpha }^{2}}|$$

It is worth noting that $\frac{\partial {\xi }_{\alpha }}{\partial x}$ is already computed by molecular mechanics force field based MD programs since it is required for the assembly of ∇*V*_*α*_. Thus, the only new quantity that must be computed is $\frac{{\partial }^{2}{V}_{\alpha }}{\partial {\xi }_{\alpha }^{2}}$, which represents a negligible additional computational cost.

We note that the final functional form (10) of our preconditioner is very similar to that of Lindh *et al*.^12^, however we arrived at it from a fundamentally different perspective, which has several advantages. Lindh *et al*.‘s method was introduced for quadratic terms only, hence the force constants have to be recomputed after each optimisation step (as the equilibrium bond lengths, angles and dihedrals are set to the actual ones to obtain a positive semidefinite matrix). Our method can be considered as a generalisation of their approach, allowing arbitrary functional forms of internal coordinate dependent terms. In particular this means that the FF parameters need not be adjusted to achieve a positive semidefinite matrix, and incorporating different parameter sets is straightforward. Moreover, as it will be discussed below our perspective makes it easy to extend the preconditioner construction to new situations. Finally we mention that since the FF-based preconditioner is a sparse matrix both its construction and inversion are computationally inexpensive, which gives the possibility to utilise it even for large systems.

### Combining FF and Exp preconditioners

For molecular crystals, intermolecular interactions also play an important role. This consideration led us to combine the molecular mechanics based FF preconditioner (describing bonded interactions) with a modified Exp preconditioner (4) (tuned to describe only non-bonded interactions, i.e. interactions already treated by the FF preconditioner are omitted):

11 $${P}_{\mathrm{Exp}+{\rm{FF}}}={P}_{\mathrm{Exp}}^{{\rm{nb}}}+{P}_{{\rm{FF}}}$$

*P*_FF_ is fully specified from the chosen force field *V*_FF_. To specify ${P}_{\mathrm{Exp}}^{{\rm{nb}}}$ we first manually choose the parameters *A* and *r*_cut_ in (4) to account for the interaction between molecules^3^. The remaining parameters are computed in a similar automatic manner as described in ref.^3^, and we keep only those matrix elements of *P*_Exp_ for which the corresponding matrix element in *P*_FF_ is zero. We note that correct scaling between *P*_FF_ and *P*_Exp_ is implicitly ensured via the *μ* parameter in Eq. 3.

### Implementation details

We tested the FF and FF + Exp preconditioners on a range of optimisation and saddle point search tasks. For geometry optimisations the form of the preconditioned LBFGS method was identical to the one we describe in ref.^3^: at each iterate the search direction is given by

12 $$\begin{array}{c}{\bf{input}}\,q=\nabla f({x}_{k})\\ {s}_{k}={x}_{k}-{x}_{k-1}\\ {y}_{k}=\nabla f({x}_{k})-\nabla f({x}_{k-1})\\ {\rho }_{k}=1/{y}_{k}^{T}{s}_{k}\\ {\rm{for}}\,i=k,\ldots ,k-m\\ \,\,{\alpha }_{i}={\rho }_{i}{s}_{i}^{T}q\\ \,\,q=q-{\alpha }_{i}{y}_{i}\\ \boxed{z={P}_{k}^{-1}q}\\ {\rm{for}}\,i=k-m,\ldots ,k\\ \,\,{\beta }_{i}={\rho }_{i}{y}_{i}^{T}z\\ \,\,z=z+({\alpha }_{i}-{\beta }_{i}){s}_{i}\\ {\bf{output}}\,{p}_{k}=z\end{array}$$

with initial search direction $z={P}_{0}^{-1}\nabla f({x}_{0})$. The box in Eq. 12 indicates the single modification of the standard algorithm to obtain preconditioning. Variable *m* is the maximum number of metric corrections. The step length selection is obtained by a backtracking line-search enforcing only the Armijo condition^3^. We note that although there exist several techniques (discussed in the Introduction) that use a quasi-Newton method in combination with some approximated Hessian information, their applicability is rather limited for large systems due to the excessive computational cost. Therefore our baseline was the unpreconditioned LBFGS method and where it was necessary we also compared our method to other preconditioning based techniques.

For saddle point search tasks we slightly modified the superlinearly converging dimer method^18^. Dimer methods use two copies of the system with coordinates *x*^(1)^ and *x*^(2)^ and a fixed separation length $l=\parallel {x}^{\mathrm{(1)}}-{x}^{\mathrm{(2)}}\parallel$ between them. The algorithm is usually split into two alternating steps^19^: (1) in the rotation step we fix the midpoint and rotate the endpoints to approximately align them with the lowest (negative) eigenmode of the Hessian (*v*_*k*_); (2) in the translation step we shift the dimer to maximise the energy along the dimer direction while minimising energy in all directions perpendicular to it (*p*_*k*_).

In principle, both the rotation and translation steps can be preconditioned, however, we found that in many systems preconditioning the rotation step results in a smaller spectral gap and hence slower convergence. Therefore, we chose to precondition only the translation step. Our implementation employs the conjugate gradient method using the Polak-Ribière formula:

13 $$\begin{array}{c}{\bf{i}}{\bf{n}}{\bf{p}}{\bf{u}}{\bf{t}}\,{q}_{k}=-\,(I-2{v}_{k}\otimes {v}_{k})\nabla f({x}_{k})\\ \boxed{\beta =\frac{{q}_{k}^{T}{P}_{k}^{-1}({q}_{k}-{q}_{k-1})}{{q}_{k-1}^{T}{P}_{k}^{-1}{q}_{k-1}}}\\ {s}_{k}={q}_{k}+\beta {s}_{k-1}\\ {\bf{o}}{\bf{u}}{\bf{t}}{\bf{p}}{\bf{u}}{\bf{t}}\,\boxed{{P}_{k}=\frac{{P}^{-1}{s}_{k}}{{s}_{k}^{T}{P}^{-1}{s}_{k}}}\end{array}$$

and using the initial iterate *s*_0_ = −(*I* − 2*v*_0_ ⊗ *v*_0_)∇*f*(*x*_0_). Again, boxed steps are the modifications needed to the original method to achieve preconditioning. For computing the step length we used the trust region radius approach suggested by Kästner and Sherwood^18^, with acceptance criterion based on the projection of the gradient of the actual step.

For molecules in gas phase *V*_FF_ is invariant under rotations and translations, hence *P*_FF_ will be at least six fold degenerate with zero eigenvalues for any configuration of the molecule corresponding to the three translational and three rotational degrees of freedom. While these degrees of freedom could in principle be fixed we found that a straightforward solution is to simply regularise the preconditioner by replacing it with $P\to P+cI$. We found that good generic values for *c* are 0.1 and 1.0 eV/Å^2^ for geometry optimisations and saddle point search, respectively.

### Model systems and potentials

#### Organic molecules in gas phase

Three potential energy surfaces were investigated for geometry optimisations: semiempirical PM6^20^, DFT^21,22^ and MP2^23^. For the DFT potential we used the the BLYP exchange-correlation functional^24,25^ with the DZVP-MOLOPT basis set^26^ and plane wave cutoff of 480 Ry within the Gaussian and plane waves method (GPW) approach^27^ and Goedecker-Teter-Hutter (GTH) pseudopotentials^28^. For the MP2 calculations the 6–31G** basis set was applied.

In the case of geometry optimisations on the PM6 surface we compared three different force fields from which we constructed the preconditioners: the force field of Lindh *et al*. (LFF)^12^, the universal force field (UFF)^29^ and the generalised Amber force field (GAFF)^30^.

Initial configurations were taken from 0.5 ns long molecular dynamics (MD) simulations at 300 K.

For transition state search we selected 7 examples (with their initial configurations) from the benchmark of Baker and Chan^31^ and three additional systems whose initial configurations were taken from 0.5 ns long MD simulations at 300 K. The computations were performed on the semiempirical PM6 surface^20^.

#### Molecular crystals

We compared four different optimisation schemes (unpreconditioned, only FF-based, only Exp-based and Exp + FF-based preconditioners) on five organic molecular crystals (systems XVIII to XXII) whose initial geometries were taken from the Organic Crystal Structure Prediction competition of the Cambridge Crystallographic Data Centre^32,33^. We used a DFT potential energy surface with the PBE exchange-correlation functional^34^ with a plane wave basis set using a cutoff energy of 800 eV and ultrasoft pseudopotentials^35^.

#### Material systems

We also tested how the force field based preconditioner works on two material systems compared to the exponential preconditioner. We examined the unpreconditioned and preconditioned geometry optimisation for bulk silicon and vacancy with varying system size. The potential energy surface was the screened Tersoff potential^36,37^ and we used the universal force field (UFF)^29^ for building the FF-based preconditioner matrix. The bulk systems were built by using the *bulk* function of ASE^38^ with default lattice constants while for the corresponding vacancy systems a silicon atom was removed. Initial configurations were obtained by applying a random displacement on the atomic positions using normal distribution with standard deviation of 0.05 Å.

Next we considered bulk tungsten and a single interstitial site in bulk tungsten, also using different system sizes. The potential energy surface was a machine learning based Gaussian Approximation Potential (GAP) reproducing the quality of DFT (with PBE functional)^39^. The preconditioner in this case was based on a simple Embedded Atom Method (EAM) potential^40,41^. Initial configurations were obtained in a similar way as the bulk silicon ones but this time a standard deviation of 0.15 Å was applied.

#### Software

For the semiempirical PM6 method AmberTools16^42^ was used. The MP2 potential surface was generated using MOLPRO^13,43^. The screened Tersoff potential was provided by Atomistica^44^. The DFT potentials with the BLYP and PBE functionals were provided by CP2K^45^ and CASTEP^46^, respectively, using the QUIP interface^47^. The GAP model was called via QUIP^47^.

In all cases the geometry optimisation was performed within ASE^38^. The other software packages were only used to compute the energy and gradient of the configurations. A Python implementation of the FF and Exp + FF preconditioners with several potential forms of nonbonded terms is available within ASE^38,48^ (https://gitlab.com/molet/ase).

Initial structures of molecules and molecular crystals as well as the generating Python codes for the starting geometry of material systems are provided as Supplementary Data.

### Accession codes

Python implementation of the preconditioners can be found here: [https://gitlab.com/molet/ase].

## Results

### Organic molecules in gas phase

**Figure 1:**
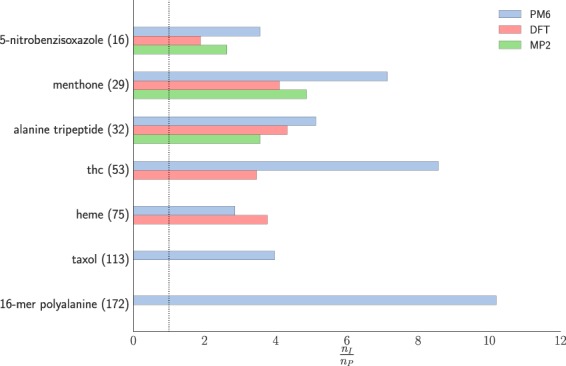
Computational saving of FF/GAFF preconditioner over unpreconditioned LBFGS optimisations for geometry optimisation of molecular systems in gas phase on three model potentials.

We investigated several organic molecules’ geometry optimisations with (FF) and without (ID) our new FF-based preconditioner on three potential energy surfaces (PM6, DFT and MP2), using the GAFF force field for building the preconditioner. The results are shown in Table 1 and Fig. 1. The convergence criterion of the geometry optimisations was $\parallel \nabla E{\parallel }_{\infty }={10}^{-3}$ eV Å^−1^ for DFT and MP2 surfaces while for the relatively inexpensive PM6 potential we applied a slightly tighter threshold of $\parallel \nabla E{\parallel }_{\infty }={10}^{-4}$ eV Å^−1^. Depending on the system and underlying potential we can observe a 4–10 fold decrease in the required number of optimisation steps using our preconditioner.

**Table 1:** Total number of function/gradient calls of geometry optimisation for organic molecules in gas phase using conventional (ID) and FF-based preconditioned (FF) LBFGS method on three different quantum chemistry surfaces. Convergence threshold was $\parallel \nabla E{\parallel }_{\infty }={10}^{-4}$ eV Å^−1^ for PM6 and $\parallel \nabla E{\parallel }_{\infty }={10}^{-3}$ eV Å^−1^ for DFT and MP2 potentials, respectively.

System (# of atoms)	PM6		DFT(BLYP)			MP2/6–31 G**		
	ID	FF/GAFF	ID	FF/GAFF	ID	FF/GAFF	FF/LFF	Lindh
5-nitrobenzisoxazole (16)	89	25	119	63	71	27	31	32
menthone (29)	207	29	197	48	107	22	31	38
alanine tripeptide (32)	395	77	536	124	210	59	47	67
thc (53)	720	84	239	69				
heme (75)	500	175	358	95				
taxol (113)	1662	419						
16-mer polyalanine (172)	3549	348

**Figure 2:**
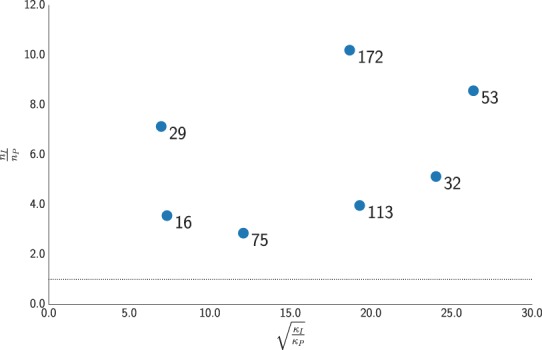
Correlation between the computational saving and the condition number ratio at potential energy minima of molecular systems in gas phase on PM6 potential.

For PM6 only, to highlight the correlation between performance gain and ill-conditioning, we also computed the ratio between the condition numbers for the unpreconditioned and preconditioned Hessians, *κ*_*I*_/*κ*_*P*_, at the minima, where

14 $${\kappa }_{P}=\frac{{\lambda }_{P}^{max}}{{\lambda }_{P}^{min}}=\frac{\mathop{max}\limits_{{u}^{T}Pu=1}{u}^{T}Hu}{\mathop{min}\limits_{{u}^{T}Pu=1}{u}^{T}\mathop{H}\limits^{ \sim }u}$$

where $\tilde{H}$ is a modified Hessian where the zero eigenvalue due to symmetries are removed. In Fig. 2 we observe that the computational saving is more strongly correlated to the condition number ratio than to the system size.

**Figure 3:**
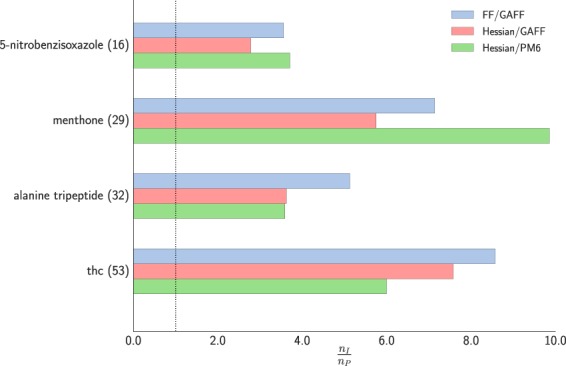
Computational gain of different optimisation algorithms compared to the unpreconditioned LBFGS method for geometry optimisation of molecular systems in gas phase on PM6 potential.

For the three smallest systems and the PM6 surface only we also compared our FF-based preconditioner against using the exact Hessian of the model potential (Hessian/GAFF) and a finite-difference Hessian of PM6 (Hessian/PM6) as preconditioners for LBFGS method. Zero eigenvalues of the Hessian matrices were shifted to a moderate positive number to avoid numerical instabilities. The results are collected in Table 2 and Fig. 3. Our FF-based preconditioner clearly outperforms both of these variants.

**Table 2:** Comparison of total number of function/gradient calls of geometry optimisation of different optimisation algorithms for minimisation of small organic molecules on PM6 surface: unpreconditioned LBFGS (ID), FF-based preconditioned LBFGS (FF/GAFF), FF-Hessian based preconditioned LBFGS (Hessian/GAFF), PM6-Hessian based preconditioned LBFGS (PM6/GAFF). Convergence threshold was $\parallel \nabla E{\parallel }_{\infty }={10}^{-4}$ eV Å^−1^ for all cases.

System (# of atoms)	LBFGS			
	ID	FF/GAFF	Hessian/GAFF	Hessian/PM6
5-nitrobenzisoxazole (16)	89	25	32	24
menthone (29)	207	29	36	21
alanine tripeptide (32)	395	77	109	110
thc (53)	720	84	95	120

**Figure 4:**
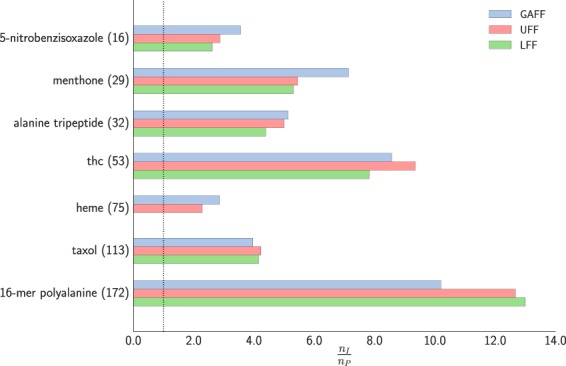
Computational gain of the FF-based preconditioner using different force fields for geometry optimisation of molecular systems in gas phase on PM6 potential.

We also examined the effect of using different force fields from which to construct the FF-based preconditioner. Beside the GAFF force field, we investigated two general force fields: the universal force field (UFF)^29^ and a general force field introduced by Lindh *et al*. (LFF)^12^. The results shown in Table 3 and Fig. 4 indicate that there is no significant difference between the three force fields. We only mention that the LFF force-field includes all possible 2, 3 and 4–body interactions^12^, resulting in a dense preconditioner matrix, which for larger systems and an efficient potential energy surface could become a performance bottleneck. By contrast, the preconditioners based on GAFF or UFF are sparse, hence their cost scales linearly with system size.

**Table 3:** Comparison of the effect of different force field based preconditioners for the geometry optimisation of organic molecules in gas phase (total number function/gradient calls). Convergence threshold was $\parallel \nabla E{\parallel }_{\infty }={10}^{-4}$ eV Å^−1^ for all cases.

System (# of atoms)	ID	FF/GAFF	FF/UFF	FF/LFF
5-nitrobenzisoxazole (16)	89	25	31	34
menthone (29)	207	29	39	39
alanine tripeptide (32)	395	77	79	90
thc (53)	720	84	77	92
heme (75)	500	175	219	n.a.
taxol (113)	1662	419	393	400
16-mer polyalanine (172)	3549	348	280	273

**Figure 5:**
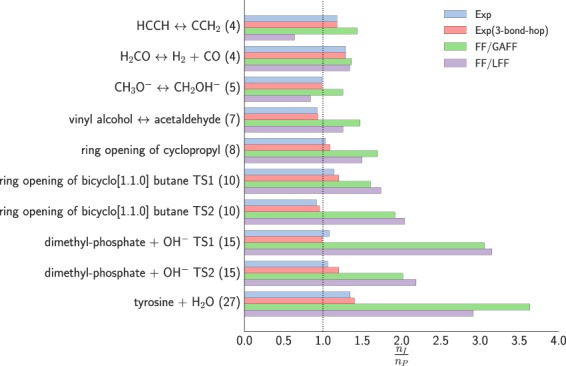
Computational gain of the Exp and FF-based preconditioners compared to the unpreconditioned superlinearly converging dimer method for molecular systems in gas phase on PM6 potential.

Finally, we tested how the different preconditioners perform when applied to transition state search, comparing again against ID (no preconditioning) and against the Exp preconditioner (with default parameter set and *μ* = 1; unlike LBFGS, CG is invariant under rescaling of *μ*). The results are collected in Table 4 and Fig. 5. Overall the Exp preconditioner does not improve significantly over ID. We experimented with different parameters, e.g., adding connectivity information up to the 4-body interaction, but observed no improvements. Both FF-based preconditioners are again comparable and yield a much improved convergence even for these relatively small systems. For instance, the gain is already 2–3-fold for dimethyl-phosphate and tyrosine hydrolyses.

**Table 4:** Number of steps of translations (and total number of function and gradient calls in parentheses) of saddle searches using different preconditioned variants of superlinearly converging dimer method. Convergence threshold was $\parallel \nabla E{\parallel }_{\infty }={10}^{-4}$ eV Å^−1^ for all cases.

System (# of atoms)	ID	Exp	FF/GAFF	FF/LFF
HCCH ↔ CCH_2_ (4)	20 (79)	17 (67)	14 (55)	32 (124)
H_2_ CO ↔ H_2_ + CO (4)	24 (94)	19 (73)	18 (69)	18 (70)
CH_3_ O^−^ ↔ CH_2_ OH^−^ (5)	18 (69)	18 (70)	14 (55)	22 (82)
vinyl alcohol ↔ acetaldehyde (7)	58 (227)	62 (245)	39 (154)	46 (179)
ring opening of cyclopropyl (8)	52 (205)	51 (199)	31 (121)	35 (137)
ring opening of bicyclo[1.1.0] butane TS 1 (10)	87 (332)	77 (291)	56 (207)	52 (191)
ring opening of bicyclo[1.1.0] butane TS 2 (10)	66 (259)	72 (282)	35 (135)	33 (127)
dimethyl-phosphate + OH^−^ TS 1 (15)	395 (1541)	361 (1425)	127 (504)	123 (489)
dimethyl-phosphate + OH^−^ TS 2 (15)	355 (1379)	329 (1302)	172 (683)	159 (631)
tyrosine + H_2_O (27)	531 (2120)	396 (1574)	147 (583)	185 (728)

### Molecular crystals

**Figure 6:**
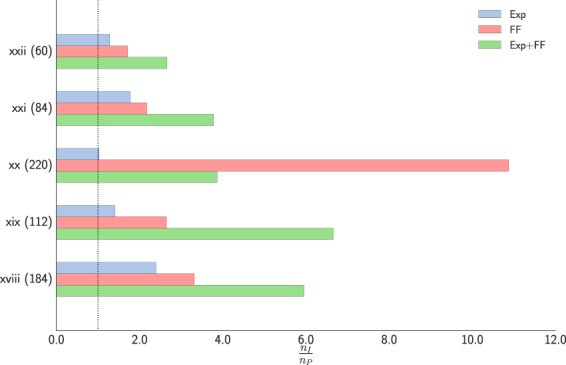
Computational gain of the Exp, FF and Exp + FF preconditioned over unpreconditioned LBFGS for geometry optimisation of molecular crystals on a DFT potential.

We compared geometry optimisation with fixed unit cells using LBFGS, preconditioned with ID (unpreconditioned), Exp^3^ and FF (GAFF force field). For Exp the nearest neighbour distance (*r*_nn_) in Eq. 4 was calculated from the initial structure, we specified *r*_cut_ = 2*r*_nn_ and *A* = 3.0. In addition, we also employed the Exp + FF preconditioner as defined in (11). The results for different molecular crystals are shown in Table 5 and Fig. 6. As expected, Exp reduces the number of optimisation steps compared to ID, although the improvement is significantly smaller for molecular crystals that for material systems^3^. Interestingly, FF alone already leads to a significant speed-up over both ID and Exp even though the inter-molecular interaction is not captured well. This indicates that for molecular crystal optimisations preconditioning based on specific intramolecular information is crucial. The most successful method was the Exp + FF combination, which leads to a 3-7 fold speed up even for these relatively small test systems.

**Table 5:** Total number of function/gradient calls of geometry optimisation of molecular crystals using different preconditioning strategies. Convergence criterion was $\parallel \nabla E{\parallel }_{\infty }={1.0}^{-3}$ eV Å^−1^.

System (# of atoms)	ID	Exp	FF/GAFF	Exp + FF/GAFF
xxii (60)	77	60	45	29
xxi (84)	291	164	134	77
xx (220)	174	169	16	45
xix (112)	193	137	73	29
xviii (184)	232	97	70	39

### Material systems

Finally, it is also interesting to investigate how an FF-based preconditioner compares against the Exp preconditioner for material systems, where Exp performs very well^3^. We tested geometry optimisation of bulk silicon and a vacancy in bulk silicon with perturbed initial conditions, with increasing system size. The screened Tersoff potential was used as the potential energy, while the FF preconditioner was constructed from UFF. The results are shown in Table 6. In both cases, the FF-based preconditioner yields a clear further speed-up over Exp for both systems.

**Table 6:** Total number of function/gradient calls geometry optimisation steps of bulk silicon and a bulk silicon vacancy using different preconditioning strategies. Convergence criterion was $\parallel \nabla E{\parallel }_{\infty }={1.0}^{-3}$ eV Å^−1^.

System (# of atoms)	Bulk geometry optimisation			Vacancy geometry optimisation		
	ID	Exp	FF/UFF	ID	Exp	FF/UFF
2 × 2 × 2 (64)	32	17	10	34	15	9
4 × 4 × 4 (512)	63	18	10	57	16	9
8 × 8 × 8 (4096)	105	21	13	96	17	11
16 × 16 × 16 (32768)	147	35	21	142	19	11

Another test system was bulk tungsten and a single interstitial site in bulk tungsten with perturbed initial conditions and different system sizes. The potential energy surface was provided by a GAP model that was trained on DFT data. The preconditioner was based on a simple EAM potential:

15 $${V}_{{\rm{E}}{\rm{A}}{\rm{M}}}=\sum _{i}[\frac{1}{2}\sum _{j\ne i}{\rm{\Phi }}({r}_{ij})+F(\sum _{j\ne i}\rho ({r}_{ij}))]$$

where Φ(*r*_*ij*_) is a pair potential, *F* is the embedding function and *ρ*(*r*_*ij*_) is the electron charge density contribution from atom *j* to atom *i*. Based on Eq. 10 our FF-based preconditioner was defined as ${P}_{{\rm{F}}{\rm{F}}}={\sum }_{\alpha }\frac{{\rm{\partial }}{r}_{\alpha }}{{\rm{\partial }}x}\otimes \frac{{\rm{\partial }}{r}_{\alpha }}{{\rm{\partial }}x}|\frac{{{\rm{\partial }}}^{2}{V}_{{\rm{E}}{\rm{A}}{\rm{M}}}}{{\rm{\partial }}{r}_{\alpha }^{2}}|$, where *α* runs over all *ij* pairs. In the actual implementation Φ, *F* and *ρ* functions are represented by splines so computing the corresponding curvature is fairly straightforward.

The results are presented in Table 7. For both the bulk and interstitial systems the number of function/gradient calls of the unpreconditioned optimisation increases with system size while the preconditioned optimisations require almost the same number of optimisation steps to achieve the same convergence criterion.

**Table 7:** Total number of function/gradient calls for geometry optimisation of bulk tungsten and interstitial defect in bulk tungsten using different preconditioning strategies. Convergence criterion was $\parallel \nabla E{\parallel }_{\infty }={1.0}^{-3}$ eV Å^−1^.

System (# of atoms)	Bulk geometry optimisation			Interstitial geometry optimisation		
	ID	Exp	FF/EAM	ID	Exp	FF/EAM
4 × 4 × 4 (64)	21	11	9	62	42	33
8 × 8 × 8 (512)	32	12	8	72	43	36
16 × 16 × 16 (4096)	56	12	9	116	46	33

## Conclusion

We introduced a flexible preconditioner for molecular simulation based on empirical potentials that are widely implemented in popular molecular mechanical program packages. Our method, which can be considered a generalisation of Lindh *et al*.^12^, decomposes the analytic Hessian of the empirical potential and modifies individual components to ensure their positivity. An advantage of this procedure is that it avoids the computation of second derivatives of the collective variables (or internal coordinates). The preconditioner yields significant improvements (at least 2 fold, and typically 5 fold decrease in function/gradient calls compared to unpreconditioned techniques), demonstrated thoroughly on a wide range of systems including molecules in gas phase, molecular crystals and materials, using different target potential energy surfaces (empirical, semiempirical and ab initio) as well as different optimisation tasks (geometry optimisations and saddle point searches).

## Electronic supplementary material

Initial geometries

## References

[1] Liu DC, Nocedal J (1989). On the limited memory bfgs method for large scale optimization. Mathematical Programming.

[2] Bakken V, Helgaker T (2002). The efficient optimization of molecular geometries using redundant internal coordinates. The Journal of Chemical Physics.

[3] Packwood D (2016). A universal preconditioner for simulating condensed phase materials. The Journal of Chemical Physics.

[4] Fogarasi G, Zhou X, Taylor PW, Pulay P (1992). The calculation of ab initio molecular geometries: efficient optimization by natural internal coordinates and empirical correction by offset forces. Journal of the American Chemical Society.

[5] Pulay P, Fogarasi G (1992). Geometry optimization in redundant internal coordinates. The Journal of Chemical Physics.

[6] Baker J (1993). Techniques for geometry optimization: A comparison of cartesian and natural internal coordinates. Journal of Computational Chemistry.

[7] Baker J, Kessi A, Delley B (1996). The generation and use of delocalized internal coordinates in geometry optimization. The Journal of Chemical Physics.

[8] Peng C, Ayala PY, Schlegel HB, Frisch MJ (1996). Using redundant internal coordinates to optimize equilibrium geometries and transition states. Journal of Computational Chemistry.

[9] Eckert F, Pulay P, Werner H-J (1997). Ab initio geometry optimization for large molecules. Journal of Computational Chemistry.

[10] Császár P, Pulay P (1984). Geometry optimization by direct inversion in the iterative subspace. Journal of Molecular Structure.

[11] Vogel S, Fischer TH, Hutter J, Lüthi HP (1993). Third-order methods for molecular geometry optimizations. International Journal of Quantum Chemistry.

[12] Lindh R, Bernhardsson A, Karlström G, Malmqvist P-A (1995). On the use of a hessian model function in molecular geometry optimizations. Chemical Physics Letters.

[13] Werner H-J, Knowles PJ, Knizia G, Manby FR, Schütz M (2012). Molpro: a general purpose quantum chemistry program package. WIREs Comput Mol Sci.

[14] Neese F (2012). The orca program system. Wiley Interdisciplinary Reviews: Computational Molecular Science.

[15] Aidas K (2014). The dalton quantum chemistry program system. Wiley Interdisciplinary Reviews: Computational Molecular Science.

[16] Dovesi R (2018). Quantum-mechanical condensed matter simulations with crystal. Wiley Interdisciplinary Reviews: Computational Molecular Science.

[17] Bitzek E, Koskinen P, Gähler F, Moseler M, Gumbsch P (2006). Structural relaxation made simple. Phys. Rev. Lett..

[18] Kästner J, Sherwood P (2008). Superlinearly converging dimer method for transition state search. The Journal of Chemical Physics.

[19] Henkelman G, Jónsson H (1999). A dimer method for finding saddle points on high dimensional potential surfaces using only first derivatives. The Journal of Chemical Physics.

[20] Stewart JJP (2007). Optimization of parameters for semiempirical methods v- Modification of nddo approximations and application to 70 elements. Journal of Molecular Modeling.

[21] Hohenberg P, Kohn W (1964). Inhomogeneous electron gas. Phys. Rev..

[22] Kohn W, Sham LJ (1965). Self-consistent equations including exchange and correlation effects. Phys. Rev..

[23] Møller C, Plesset MS (1934). Note on an approximation treatment for many-electron systems. Phys. Rev..

[24] Becke AD (1988). Density-functional exchange-energy approximation with correct asymptotic behavior. Phys. Rev. A.

[25] Lee C, Yang W, Parr RG (1988). Development of the colle-salvetti correlation-energy formula into a functional of the electron density. Phys. Rev. B.

[26] VandeVondele J, Hutter J (2007). Gaussian basis sets for accurate calculations on molecular systems in gas and condensed phases. The Journal of Chemical Physics.

[27] Lippert G, Hutter J, Parrinello M (1997). A hybrid gaussian and plane wave density functional scheme. Molecular Physics.

[28] Goedecker S, Teter M, Hutter J (1996). Separable dual-space gaussian pseudopotentials. Phys. Rev. B.

[29] Rappe AK, Casewit CJ, Colwell KS, Goddard WA, Skiff WM (1992). Uff, a full periodic table force field for molecular mechanics and molecular dynamics simulations. Journal of the American Chemical Society.

[30] Wang J, Wolf RM, Caldwell JW, Kollman PA, Case DA (2004). Development and testing of a general amber force field. Journal of Computational Chemistry.

[31] Baker J, Chan F (1996). The location of transition states: A comparison of cartesian, z-matrix, and natural internal coordinates. Journal of Computational Chemistry.

[32] Bardwell DA (2011). Towards crystal structure prediction of complex organic compounds–a report on the fifth blind test. Acta Crystallographica Section B.

[33] Reilly AM (2016). Report on the sixth blind test of organic crystal structure prediction methods. Acta Crystallographica Section B.

[34] Perdew JP, Burke K, Ernzerhof M (1996). Generalized gradient approximation made simple. Phys. Rev. Lett..

[35] Vanderbilt D (1990). Soft self-consistent pseudopotentials in a generalized eigenvalue formalism. Phys. Rev. B.

[36] Tersoff J (1986). New empirical model for the structural properties of silicon. Phys. Rev. Lett..

[37] Pastewka L, Klemenz A, Gumbsch P, Moseler M (2013). Screened empirical bond-order potentials for si-c. Phys. Rev. B.

[38] Bahn SR, Jacobsen KW (2002). An object-oriented scripting interface to a legacy electronic structure code. Computing in Science Engineering.

[39] Szlachta WJ, Bartók AP, Csányi G (2014). Accuracy and transferability of gaussian approximation potential models for tungsten. Phys. Rev. B.

[40] Daw MS, Baskes MI (1984). Embedded-atom method: Derivation and application to impurities, surfaces, and other defects in metals. Phys. Rev. B.

[41] Zhou XW, Johnson RA, Wadley HNG (2004). Misfit-energy-increasing dislocations in vapor-deposited cofe/nife multilayers. Phys. Rev. B.

[42] Case, D. A. *et al*. Amber 16. Tech. Rep., University of California, San Francisco, http://www.ambermd.org (2016).

[43] Werner, H.-J. *et al*. Molpro, version 2015.1, a package of ab initio programs, https://www.molpro.net (2015).

[44] Pastewka, L. Atomistica: interatomic potentials library, http://www.atomistica.org.

[45] VandeVondele J (2005). Quickstep: Fast and accurate density functional calculations using a mixed gaussian and plane waves approach. Computer Physics Communications.

[46] Segall MD (2002). First-principles simulation: ideas, illustrations and the castep code. Journal of Physics: Condensed Matter.

[47] Csányi, G. *et al*. Expressive programming for computational physics in fortran 95+. *IoP Comput. Phys. Newsletter**Spring*, http://www.libatoms.org (2007).

[48] Larsen AH (2017). The atomic simulation environment—a python library for working with atoms. Journal of Physics: Condensed Matter.

