# Supplementary material for: Preconditioners for the geometry optimisation and saddle point search of molecular systems
Source: Sci Rep. 2018 Sep 18;8:13991. doi: 10.1038/s41598-018-32105-x (PMC6143621; doi:10.1038/s41598-018-32105-x)
Supplement: Supplementary file 1 — Initial geometries [file 41598_2018_32105_MOESM1_ESM.zip › Supplementary_Information/Supplementary_Information.pdf]

Supplementary Information for: Preconditioners for the geometry optimisation and saddle point search of molecular systems

Letif Mones[1,2,*], **Gábor Csányi**[2], **and Christoph Ortner**[1]

[1]Mathematics Institute, University of Warwick, Zeeman Building, Coventry, CV4 7AL, United Kingdom
[2]Engineering Laboratory, University of Cambridge, Trumpington Street, Cambridge, CB2 1PZ, United Kingdom
[*]lam81@cam.ac.uk

Initial geometries

For molecular and molecular crystal systems we provide the explicit initial structures in extended XYZ format (that includes the lattice information as well), while for material systems the generating Python codes are given. Systems and their corresponding geometries / generating codes are listed in Table 1.

System	extended XYZ file / generating Python code
5-nitrobenzisoxazole	`5-nitrobenzisoxazole.exyz`
menthone	`menthone.exyz`
alanine tripeptide	`trialanine.exyz`
thc	`thc.exyz`
heme	`heme.exyz`
taxol	`taxol.exyz`
16-mer polyalanine	`ace_ala16_nme.exyz`
HCCH $\leftrightarrow$ CCH_2	`hcch.exyz`
H_2CO $\leftrightarrow$ H_2 + CO	`h2co.exyz`
CH_3O^- $\leftrightarrow$ CH_2OH^-	`ch3o.exyz`
vinyl alcohol $\leftrightarrow$ acetaldehyde	`ch2choh.exyz`
ring opening of cyclopropyl	`cyclopropyl.exyz`
ring opening of bicyclo[1.1.0] butane TS 1	`bicyclobutane_ts1.exyz`
ring opening of bicyclo[1.1.0] butane TS 2	`bicyclobutane_ts2.exyz`
dimethyl-phosphate + OH^- TS 1	`dmp_oh_ts1.exyz`
dimethyl-phosphate + OH^- TS 2	`dmp_oh_ts2.exyz`
tyrosine + H_2O	`tyrosine_water.exyz`
xxii	`xxii.exyz`
xxi	`xxi.exyz`
xx	`xx.exyz`
xix	`xix.exyz`
xviii	`xviii.exyz`
silicon bulk systems	`si_bulk_systems.py`
silicon vacancy systems	`si_vacancy_systems.py`
tungsten bulk systems	`w_bulk_systems.py`
tungsten interstitial systems	`w_interstitial_systems.py`

Table 1. List of initial geometries provided by extended XYZ format or generating Python code.
